# Genome-wide identification of *CLE gene* family and their potential roles in bolting and fruit bearing in cucumber (*Cucumis sativus* L.)

**DOI:** 10.1186/s12870-021-02900-2

**Published:** 2021-03-19

**Authors:** Nannan Qin, Yang Gao, Xiaojing Cheng, Yang Yang, Jiang Wu, Jinyao Wang, Sen Li, Guoming Xing

**Affiliations:** College of Horticulture, Shanxi Agricultural University, Taigu, China

**Keywords:** CLE peptides, Cucumber, Gene family, Phylogenetic analysis

## Abstract

**Background:**

Signal peptides are essential for plant growth and development. In plants, biological processes including cell-cell communication, cellular proliferation and differentiation, cellular determination of self-incompatibility, and defensive responses, all depend heavily on peptide-signaling networks such as CLE (CLAVATA3/Embryo surrounding region-related). The CLEs are indispensable in different periods of plant growth and development, especially in maintaining the balance between proliferation and differentiation of stem cells in various meristematic tissues.

The working system of *CLE* genes in cucumber, an important economical vegetable (*Cucumis sativus* L.), has not been fully studied yet. The distributional patterns of chromosome-level genome assembly in cucumber provide a fundamental basis for a genome-wide comparative analysis of *CLE* genes in such plants.

**Results:**

A total of 26 individual *CLE* genes were identified in Chinese long ‘9930’ cucumber, the majority of which belong to unstable short alkaline and hydrophilic peptides. A comparative analysis showed a close relationship in the development of CLE genes among *Arabidopsis thaliana*, melon, and cucumber. Half of the exon-intron structures of all *CsCLEs* genes are single-exon genes, and motif 1, a typical CLE domain near the C-terminal functioning in signal pathways, is found in all cucumber CLE proteins but CsCLE9. The analysis of CREs (Cis-Regulatory Elements) in the upstream region of the 26 cucumber *CLE* genes indicates a possible relationship between *CsCLE* genes and certain functions of hormone response elements. Cucumber resulted closely related to *Arabidopsis* and melon, having seven and 15 orthologous *CLE* genes in *Arabidopsis* and melon, respectively. Additionally, the calculative analysis of a pair of orthologous genes in cucumber showed that as a part of the evolutionary process, *CLE* genes are undergoing a positive selection process which leads to functional differentiation. The specific expression of these genes was vigorous at the growth and development period and tissues. Cucumber gene *CLV3* was overexpressed in *Arabidopsis*, more than half of the transformed plants in T1 generation showed the phenomena of obvious weakness of the development of growing point, no bolting, and a decreased ability of plant growth. Only two bolted strains showed that either the pod did not develop or the pod was short, and its development was significantly inferior to that in the wild type.

**Conclusions:**

In this study, 26 *CLE* genes were identified in Chinese long ‘9930’ cucumber genome. The *CLE* genes were mainly composed of alkaline hydrophilic unstable proteins. The genes of the *CLE* family were divided into seven classes, and shared close relationships with their homologs in *Arabidopsis* and melon. The specific expression of these genes was evaluated in different periods of growth and tissue development, and *CLV3*, which the representative gene of the family, was overexpressed in *Arabidopsis*, suggesting that it has a role in bolting and fruit bearing in cucumber.

**Supplementary Information:**

The online version contains supplementary material available at 10.1186/s12870-021-02900-2.

## Background

Signal peptides are short peptide chains that guide the transfer of newly synthesized proteins to the secretory pathway, and are generally 5–30 amino acids in length. Plant small signaling peptides play important roles in eukaryotic growth and development process, for instance, stress response [1–3], apical meristem maintenance [4–6], vascular bundle development [7], stomata development [8], reproductive growth [9], cell division [10], and mineral element absorption [11], as well as cell-cell communication, self-incompatibility determination and other molecular-level roles [12]. The CLAVATA3(CLV3)/EMBRYO SURROUNDING REGION (CLE) peptides, a better studied class of signal peptide molecules, consist of 12 or 13 amino acids, including hydroxylated proline residues that may or may not contain sugar modifications, and function in a non-cell-autonomous fashion. They function in the extracellular space as intercellular signaling molecules and bind to cellular surface receptor-like proteins to transmit a signal. The CLE polypeptide hormones play a key role in many important processes of higher plant growth and development, especially in maintaining the balance between proliferation and differentiation of stem cells in different meristematic tissues [13, 14]. The CLE peptide hormones, in regulating stem meristem of transcription factors in the cell nucleus *WUS (WUSCHEL)*, promote the expression of stem cell marker genes *CLV3*, maintain stem cell properties, while the expression of *CLV3* inhibiting *WUS* transcription level promote stem cell differentiation, and form a very precise feedback control loop that controls the number of stem cells in the stem meristem [13]. In the distal root meristem, a self-regulating interaction feedback loop is also formed between the ACR4-CLE40-mediated signal and the WUS family transcription factor WOX5 to regulate the proliferation and differentiation of the distal root meristem [13, 14]. Vascular cambium, as the initial center of plant vascular phylogeny, has the characteristics of pluripotent stem cells. Hirakawa, Fisher, Turner et al. [15–17] found that there is a regulatory mechanism whereby vascular cambium cell division is regulated by the *PXY/TDR* gene expressed in the *Arabidopsis* cambium cells and the *CLE41/ TD1F* gene expressed in the phloem element differentiation inhibitory factor. Hirakawa et al. [16] found that vascular bundles of *Arabidopsis* that overexpressed *CLE41* and *CLE44* were fractured, resulting in excessive division of procambium cells and significant enlargement of vascular regions. Gene *CLE41/TDIF* expressed specifically in the phloem of *Arabidopsis* can promote the proliferation of procambium cells and inhibit the differentiation of procambium cells into conduit molecules. Except for *CLE41/44* and *CLE42*, all other CLE polypeptides, including CLV3, do not perform this function. The PXY/TDR mutation of the receptor of CLE41/44 would make the procambium cells disorientated during division, reducing the differentiated xylem and phloem cells, while retaining more undifferentiated procambium cells [15–17]. The expression level of *WUS* homologous gene *WOX4* in the procambium was shown to be regulated by *CLE41/44* [16].

Cucumber (*Cucumis sativus* L.; 2n = 2x = 14), is an economically important vegetable crop worldwide. It is consumed in the immature or mature, fresh or processed form. Cucumber genome was sequenced early in 2009 due to its small genome size (around 367 Mb) and agricultural importance, providing insight into traits such as its sex expression, disease resistance, biosynthesis of cucurbitacin, and ‘fresh green’ odor [18]. This draft genome was assembled using low-coverage Sanger sequences and high-coverage short Illumina sequences over a 70-fold genome coverage for the Chinese long inbred line ‘9930’, 72.2-fold genome coverage. For years, researchers have shown interest in whole-genome and whole-transcriptome sequencing that resulted in several versions of the cucumber genome and provided genetic information. Scaffold assemblies of three cucumber lines (Chinese long’9930′, Gy14 [19], and B10) are available so far. Mainstream genome assembly and gene annotation of cucumber cultivar Chinese long ‘9930’ have been updated to version 3. Version 3 of the cucumber genome was assembled using PacBio long reads, 10X Genomics, and high-throughput chromosome conformation capture (Hi-C) data. This accurate genome assembly, containing 174 contigs with a total length of 226.2 Mb and an N50 of 8.9 Mb, identified 1078 novel genes, including 239 tandemly-duplicated genes and 1374 full-length long terminal retrotransposons [20]. Additionally, many cucumber genome and transcriptome databases were constructed to provide user-friendly query interfaces for genomic research and breeding. In 2012, the first draft genome of melon became available. The double-haploid line DHL92 was sequenced and 375 Mb (83.3%) of melon genome has been assembled, including 27,427 protein-coding genes. Since the ancient eudicot triplication, the recent whole-genome duplications were absent in the melon lineage, which might have resulted in the increased size of melon genome compared with cucumber [21]. The genome-wide identification of *CLE* genes in melon and further comparison with cucumber homologs will help elucidate the evolutionary pattern within cucurbit species. The available cucumber genome assembly and abundant tools have accelerated genomic research, including gene family analysis through a bioinformatic approach. Genome-wide gene family analysis has been implied in numerous cucumber gene families, including MADS-box [22], SWEET [23], auxin response factor [24], lipoxygenase [25], calcium-dependent protein kinase [26], metacaspase [27], glutathione peroxidase [28], and aquaporin [29], but the specific biological and functional roles of the *CLE* genes in cucumber remain unknown.

The present study focuses on a genome-wide analysis of *CLE* genes in cucumber. We analyzed the exon-intron structure, gene phylogeny and synteny, and the tissue-specific expression of the identified *CLE* genes, as well as heterologous overexpression of cucumber *CLV3* gene in *Arabidopsis*. The objective of this study was to provide insights into the genetic network of signaling peptides in cucumber and other cucurbit crops which should in the future facilitate improvement in yield, nutritional value, and commercial quality.

## Results

### Genome-wide identification of *CLE* genes in cucumber

The genome-wide search for *CLE* genes in cucumber was done using alignment. We first downloaded the known CLE proteins of *Arabidopsis* to self-construct a CLE Markov model. By scanning all 24,317 cucumber coding proteins in the genome assembly version 3 using the designed CLE Markov model, we identified 26 CLE transcription factors (S1 Table). Manual check revealed well conserved CLE transcription factors (referred by the term ‘CsCLE’ with a serial number and sorted by the E-value of the CLE domain; Table 1). Many cucumber CLE peptides are short peptides (90 amino-acid long) except CsCLE24 (1021 amino acids), CsCLE5 (416 amino acids), and CsCLE6 (404 amino acids). The molecular weight of the cucumber CLE proteins ranged from 5293.84 (CsCLE9) to 113,323.11 (CsCLE24), which is very similar to those in *Arabidopsis*. Most cucumber CLE proteins are alkaline with a theoretical pI ranging from 7.09 (CsCLE17) to 12.11 (CsCLE10), except for one weakly acidic protein, CsCLE24 (pI = 6.52). The CsCLE4 protein is stable whereas others are unstable. The aliphatic index of cucumber CLE proteins ranged from 49.03 (CsCLE6) to 106.78 (CsCLE17), and the GRAVY index ranged from − 0.935 (CsCLE9) to 0.17 (CsCLE21).

**Table 1:** Characteristics of CLE gene family members in cucumber genome

Gene	Number of amino acids	Molecular weight	Theoretical pI	Formula	Instability index	Stability	Aliphatic index	Grand average of hydropathicity (GRAVY)	Gene ID	Protein ID	Chromosome	Start	End
CsCLE1	98	10,680.15	9.62	C468H748N136O142S4	45.68	unstable	74.69	−0.347	CsaV3_1G002320	CsaV3_1G002320.1	chr1	1,490,477	1,490,773
CsCLE2	95	10,833.66	9.23	C491H771N137O130S5	56.01	unstable	95.47	−0.064	CsaV3_1G005510	CsaV3_1G005510.1	chr1	3,589,463	3,590,462
CsCLE3	109	12,477.54	9.89	C558H873N161O149S8	69.27	unstable	66.15	−0.504	CsaV3_1G015250	CsaV3_1G015250.1	chr1	10,837,630	10,838,848
CsCLE4	110	12,200.05	8.68	C527H850N150O162S10	30.58	stable	80.55	−0.176	CsaV3_1G032100	CsaV3_1G032100.1	chr1	19,099,504	19,100,195
CsCLE5	416	45,422.36	8.55	C1974H3088N566O643S12	62.6	unstable	55.79	−0.701	CsaV3_1G038290	CsaV3_1G038290.1	chr1	24,073,345	24,074,694
CsCLE6	404	43,809.33	9.25	C1888H2967N555O626S11	61.62	unstable	49.03	−0.825	CsaV3_2G006930	CsaV3_2G006930.1	chr2	3,447,879	3,452,939
CsCLE7	87	9948.6	11.09	C426H723N141O123S5	94.63	unstable	99.66	−0.363	CsaV3_2G010680	CsaV3_2G010680.1	chr2	7,844,854	7,846,926
CsCLE8	66	7406.7	7.86	C337H518N86O90S6	57.75	unstable	76.82	0.1	CsaV3_2G011430	CsaV3_2G011430.1	chr2	8,729,651	8,729,911
CsCLE9	49	5293.84	8.04	C217H360N78O73S2	61.51	unstable	59.8	−0.935	CsaV3_2G017950	CsaV3_2G017950.1	chr2	14,938,987	14,939,136
CsCLE10	94	10,354.26	12.11	C461H778N144O122S2	58.84	unstable	97.45	−0.296	CsaV3_2G026370	CsaV3_2G026370.1	chr2	18,057,981	18,058,265
CsCLE11	197	22,244.05	10.54	C963H1557N303O296S4	82.23	unstable	70.86	−0.795	CsaV3_3G029850	CsaV3_3G029850.1	chr3	25,594,981	25,596,269
CsCLE12	87	9737.34	9.39	C433H706N120O126S4	84.37	unstable	96.32	−0.216	CsaV3_4G033240	CsaV3_4G033240.1	chr4	23,527,565	23,528,521
CsCLE13	89	9650.95	8.4	C432H676N118O129S2	60.32	unstable	74.61	−0.287	CsaV3_5G003050	CsaV3_5G003050.1	chr5	1,933,962	1,934,888
CsCLE14	80	8694.96	8.31	C391H603N113O107S3	21.9	unstable	95	0.099	CsaV3_5G003100	CsaV3_5G003100.1	chr5	1,951,210	1,951,452
CsCLE15	99	10,877.5	11.29	C496H763N141O132S2	58.3	unstable	71.11	−0.205	CsaV3_5G028390	CsaV3_5G028390.1	chr5	23,581,219	23,582,177
CsCLE16	108	11,738.69	9.69	C527H839N151O143S5	35.76	unstable	85.65	−0.193	CsaV3_5G032070	CsaV3_5G032070.1	chr5	26,044,500	26,045,876
CsCLE17	115	12,571.58	7.09	C562H903N153O163S5	63.43	unstable	106.78	0.15	CsaV3_5G033190	CsaV3_5G033190.1	chr5	26,649,447	26,649,794
CsCLE18	107	12,110.72	8.82	C536H827N157O155S5	74.14	unstable	62.99	−0.67	CsaV3_5G034340	CsaV3_5G034340.1	chr5	27,330,542	27,332,804
CsCLE19	90	10,637.26	10.27	C487H739N139O125S3	48.41	unstable	75.78	−0.359	CsaV3_5G037710	CsaV3_5G037710.1	chr5	29,863,702	29,864,814
CsCLE20	148	16,584.49	10.74	C743H1235N209O210S4	50.33	unstable	98.85	−0.307	CsaV3_6G004590	CsaV3_6G004590.1	chr6	3,923,995	3,924,868
CsCLE21	88	9875.56	10.04	C453H704N130O113S3	37.63	unstable	105.34	0.17	CsaV3_6G018750	CsaV3_6G018750.1	chr6	13,318,223	13,318,712
CsCLE22	104	11,124.83	9.64	C484H780N146O141S7	47.5	unstable	72.12	−0.3	CsaV3_6G036050	CsaV3_6G036050.1	chr6	20,010,237	20,012,042
CsCLE23	92	10,326.16	9.81	NOT SURE	65.39	unstable	73.26	−0.266	CsaV3_6G037620	CsaV3_6G037620.1	chr6	21,296,034	21,296,394
CsCLE24	1021	113,323.11	6.52	C4870H7888N1414O1612S40	47.03	unstable	72.78	−0.708	CsaV3_6G050690	CsaV3_6G050690.1	chr6	29,541,626	29,551,082
CsCLE25	80	8987.28	8.89	C411H623N113O111S2	48.13	unstable	86.75	−0.125	CsaV3_6G050700	CsaV3_6G050700.1	chr6	29,552,032	29,552,520
CsCLE26	106	11,610.31	9.19	C532H803N139O146S4	56.02	unstable	76.42	0.122	CsaV3_7G025870	CsaV3_7G025870.1	chr7	15,350,171	15,352,628

**Fig. 1:**
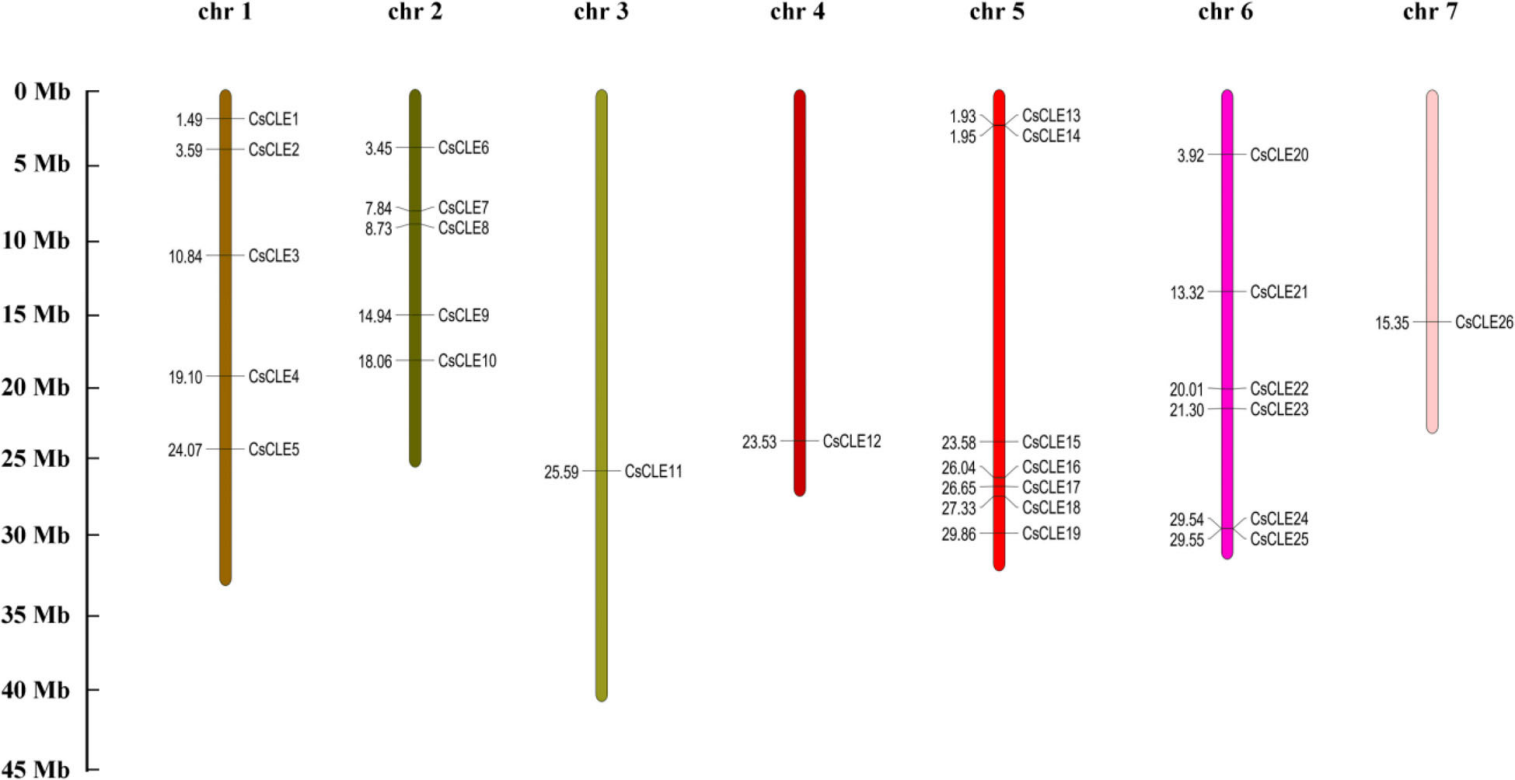
Chromosome distribution of *CLE* genes in cucumber. Different line colours represent different chromosomes and marks on them are the corresponding genes and their locations

**Fig. 2:**
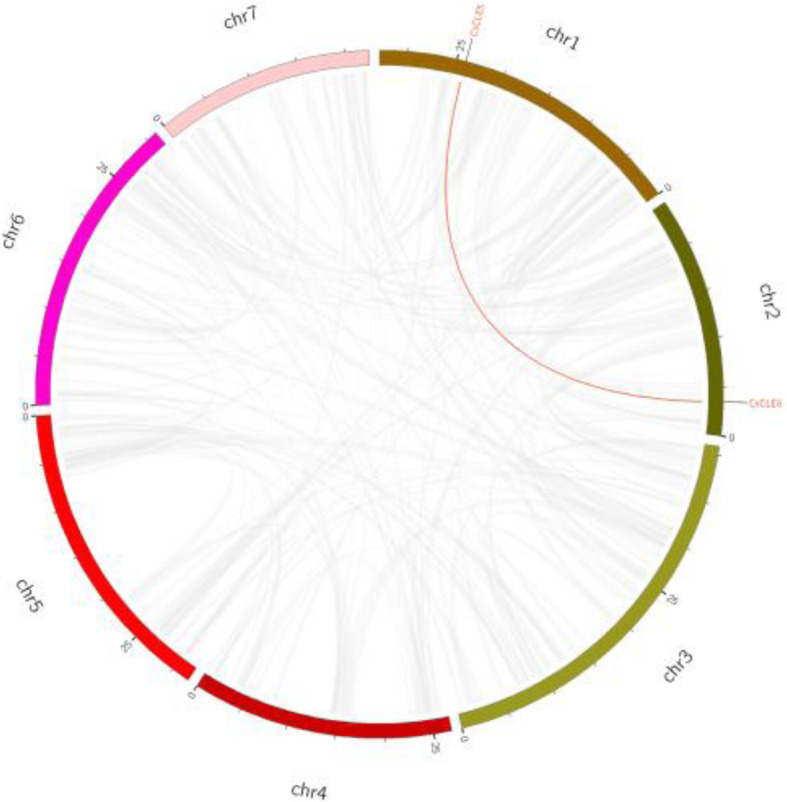
Distribution of segmental duplication *CLE* genes *CLE5* and *CLE6* in the cucumber genome. Two genes of the same segmental duplicated gene pair are labelled in red

The 26 *CLE* genes remained distributed across chromosomes, including 7 on chr5, 6 on chr6, 5 each on chr1 and chr2, and one each on chr3, chr4, and chr7 (Fig. 1). The *CLE* genes *CsCLE24* and *CsCLE25* were located extremely close with a short intergenic location of 1 kb. Besides, *CsCLE13* and *CsCLE14* were also located in close proximity; this finding indicated that these genes might be tandemly duplicated. In addition, one gene pair, *CsCLE5* and *CsCLE6*, with segmental duplication was found within the *CsCLE* family, and the identified segmental duplication gene pairs were distributed on different chromosomes in cucumber (Fig. 2).

### Phylogenetic relationships and divergence of cucumber CLE proteins

In the present study, we primarily use the genome assembly of Chinese long ‘9930’.

**Fig. 3:**
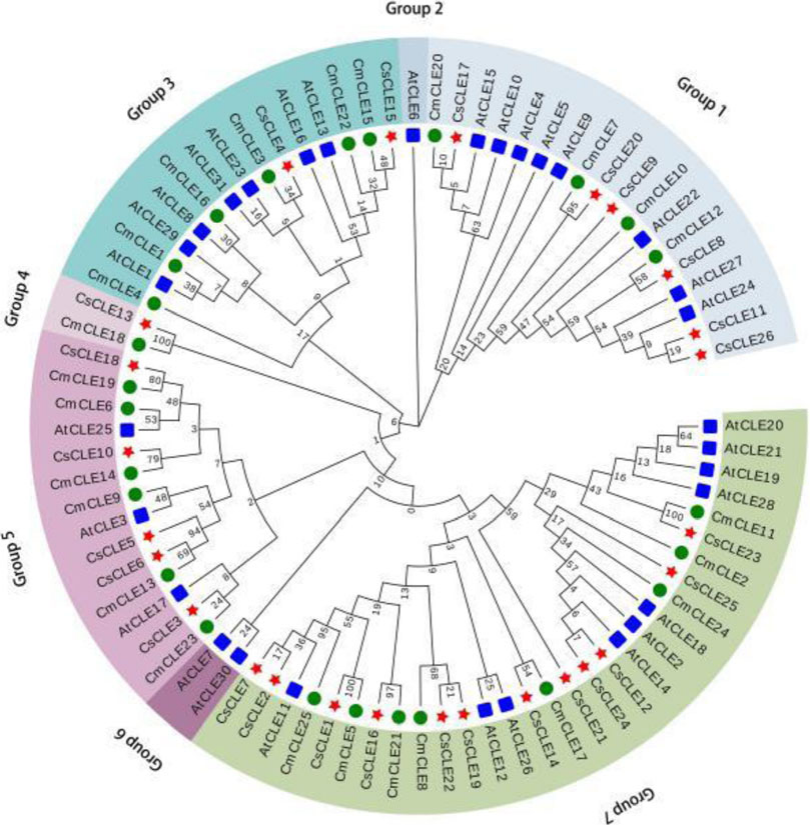
Phylogenetic analysis of *CLE* genes from *Arabidopsis*, cucumber, and melon. The analysis involved 26 cucumber CLE protein sequences, 31 *Arabidopsis* AtCLE protein sequences, and 25 melon CLE protein sequences. Red stars represent the cucumber CLE proteins; blue squares represent the *Arabidopsis* CLE proteins; and green circles represent the melon CLE proteins

Only 31 *CLE* genes were identified (S2 Table) in this report, while a previous report showed that *Arabidopsis* contained 32 *CLE* genes [30]. A total of 25 *CLE* genes were identified in melon (S3 Table) using the computational approach. To understand the evolution of *CLE* genes in Cucurbitaceae and the model plant *Arabidopsis*, we compared the genes from *Arabidopsis*, melon, and the cucumber cultivar Chinese long ‘9930’ (Fig. 3). These *CLEs* were divided based on their phylogenetic relationship into seven paraphyletic groups (Groups 1 to 7). All groups except for Group 2 and Group 6 were composed of *CLEs* from both cucumber and other plant species, indicating the close relationship between cucumber *CLEs* and those of other plants. The distribution of cucumber *CLEs* was uneven in these groups. Group 7 was the largest group with 12 members, accounting for nearly half of the cucumber *CLEs*, followed by Groups 1 and 5 containing 6 and 5 cucumber *CLEs*, respectively. The least represented subfamilies were Groups 2 and 6 with no cucumber *CLEs.*

### Gene structure of cucumber *CLE* genes

**Fig. 4:**
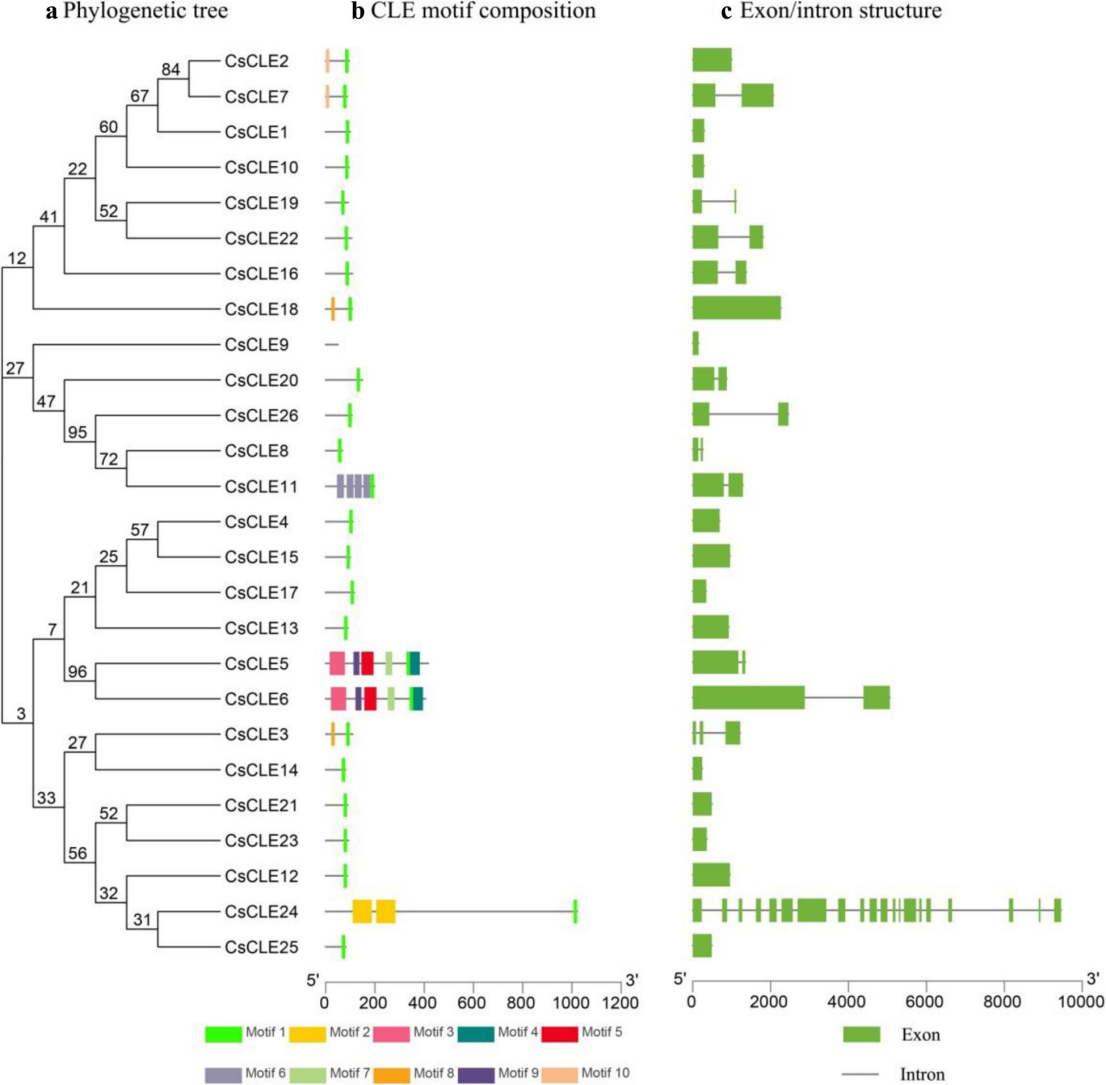
Predicted exon-intron structures of cucumber *CLEs* and conserved motifs of cucumber CLE protein. **a** The phylogenetic tree was constructed based on the full-length protein sequences of 26 cucumber CLE proteins using MEGA V6.06 software. **b** Conserved motifs in cucumber CLE proteins. The motifs, numbers 1–10, were displayed in differently coloured boxes. **c** Exon-intron structure of cucumber *CLE* genes. Exons and introns are indicated by green boxes and single lines, respectively

In order to compare the cucumber genes, their exon-intron structures were predicted (Fig. 4). The phylogenetic tree was constructed by the Maximum-Likelihood method using the conserved protein sequence of the cucumber *CLE* genes. Fourteen *CLE* genes were single-exon genes, ten were double-exon genes, and one was a triple-exon gene. The exon-intron patterns within the same phylogenetic classification group showed similarity. Cucumber gene *CLE24* was unique with its discrete coding regions split by 19 introns.

### Conserved cucumber CLE protein motifs

The MEME software was used to explore the conserved domains and motifs of the CLE proteins in cucumber. The motifs were listed from motif 1 to 10 according to the ascending E-value of the alignment (Fig. 4). Almost all cucumber CLE proteins contained motif 1, which represents a typical CLE domain near the C-terminal, except for CsCLE9. Proteins CsCLE5 and CsCLE6 were different at the C-terminal; both contained motif 4 near the C-terminal CLE domain. Protein CsCLE11 contained four tandem located motif 6 and one CLE domain, while CsCLE24 contained two motif 2 in tandem. These results indicated that the sequence characterization and biological function widely differed among cucumber CLE proteins in each branch of the phylogenetic tree. These proteins also possessed other functional domains at the N-terminal or in the middle, while their CLE domain was functional in signal pathways.

### Upstream cis-regulatory elements (CREs)

**Fig. 5:**
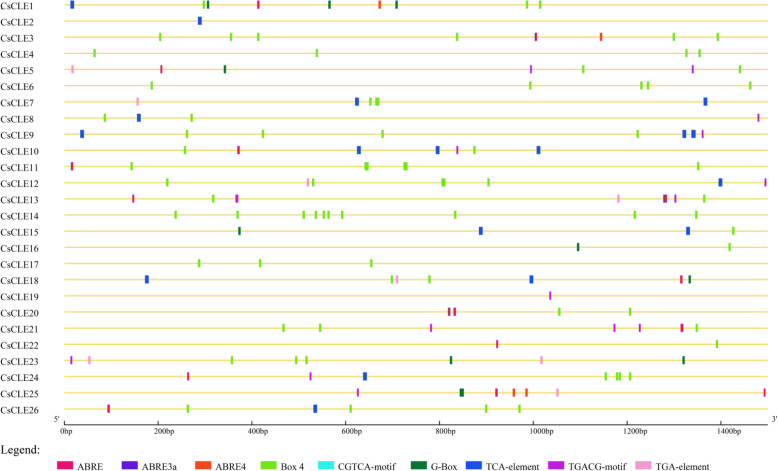
Hormone response elements upstream of cucumber *CLEs*. The elements which respond to hormones are displayed in differently coloured boxes

In the PlantCARE database, the sequence of 1500 bp in the upstream promoter was used to locate the cis-regulatory element in the upstream regulatory region. By analyzing the biological functions of CREs in the upstream region of 26 cucumber *CLE* genes, it can be concluded that most of the components other than those necessary for the normal expression of promoters, such as AT~TATA-Box and CAAT-Box, can be divided into the following four categories. 1) The first type of element is hormone response elements, such as ABRE (ABA response element), the TCA-element (salicylic acid response element), the TATC-Box (gibberellin response element), and the TGA-element (auxin response element), among others. These elements were plotted in this experiment (Fig. 5). 2) The second type of element is light-responsive elements, such as ACE, G-box, ATA-motif, LAMP-element, etc. 3). The third type of element is the meristematic expression elements. For example, HD-zip1 (elements related to mesophyll cell differentiation) and TGACG-motif (elements related to meristem development) play an important role in CLE gene regulation of plant meristem maintenance and organogenesis. 4) The fourth type is stress response elements, such as ARE, MBS, TC-rich repeats, LTR, etc., as well as other regulatory factors that play a role in metabolic regulation.

### Homologous pairs of cucumber CLE proteins

To understand the vertical descent from a single ancestral gene and duplication, a comparative analysis was performed to identify the orthologous, co-orthologous, and paralogous gene pairs in *Arabidopsis*, cucumber, and melon.

**Fig. 6:**
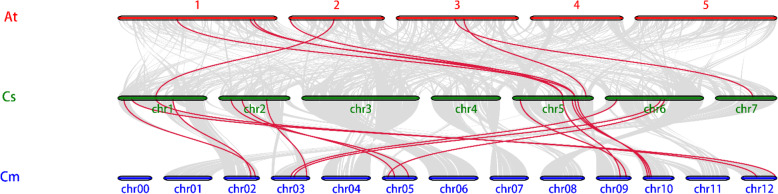
Homologous gene pairs in *Arabidopsis*, cucumber, and melon. The gray lines represent all the homologous pairs between different genomes, and the red lines represent the homologous pairs of the *CLE* gene family

The OrthoMCL software identified 24 orthologous, 23 co-orthologous, and 8 paralogous *CLE* gene pairs among *Arabidopsis*, cucumber, and melon. This homologous gene pair network was highly consistent with the phylogenetic tree, which is clearly divided into three parts. The orthologous genes between these three species are shown in Fig. 6. Out of 26 cucumber *CLE* genes, 15 orthologous genes in melon and 7 in *Arabidopsis* were found.

### Selection and divergence time

In order to investigate the selection mechanisms of *CLE* genes during evolution, we calculated Ka, Ks, and divergence time of the homologous pair *CsCLE24-CsCLE25* (S4 Table). This pair had a Ka of 1.08 and Ks of 0.82, and Ka/Ks ratio of 1.31 with a *P*-value of 0.0098, indicating this gene pair is undergoing a positive selection which led to functional differentiation.

### Expression pattern of cucumber *CLE* genes during development

**Fig. 7:**
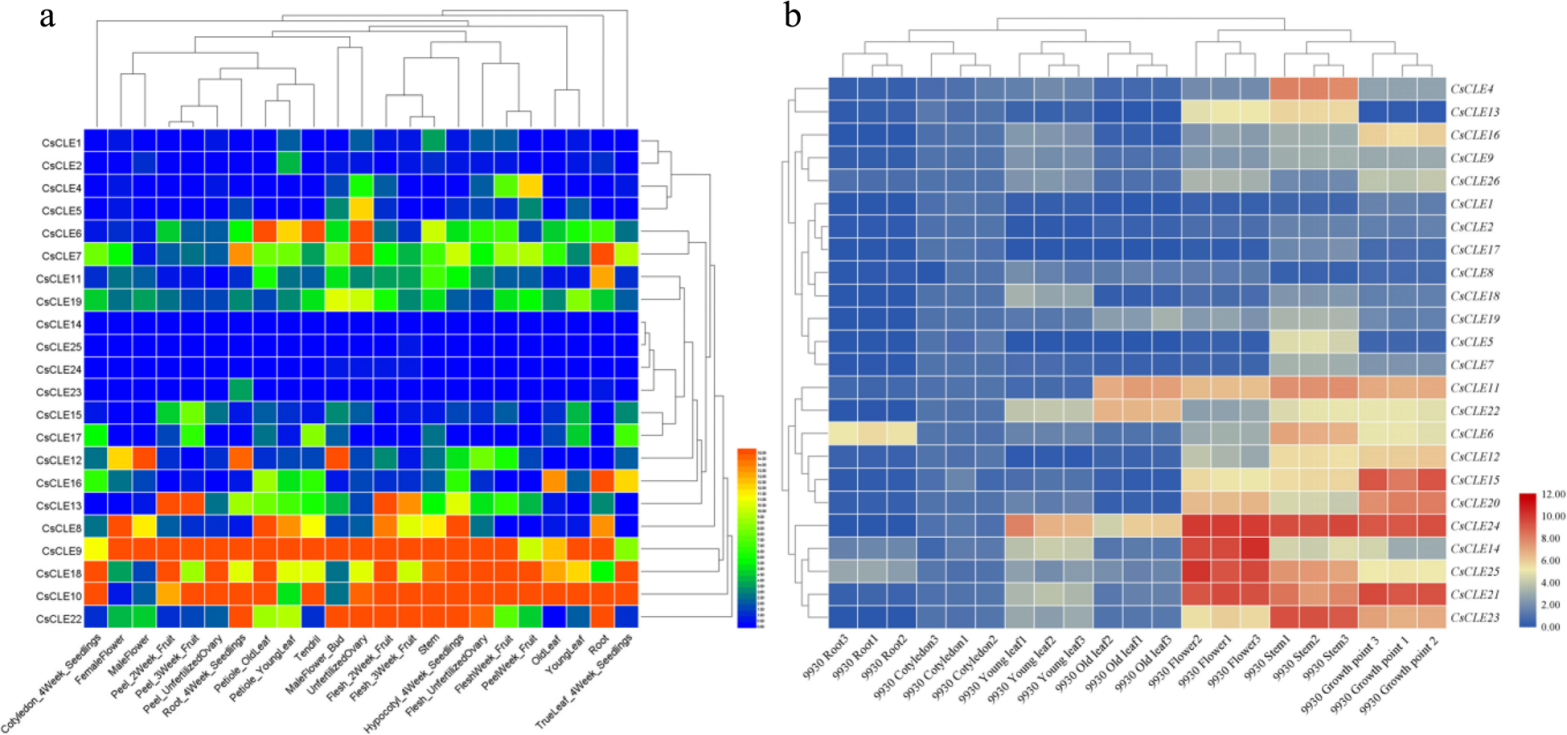
Expression pattern of cucumber *CLE* genes. **a** Heat map representing the expression profile of cucumber *CLE* genes during different development stages analyzed by RNA-seq data. **b** Heat map representing the expression profile of cucumber *CLE* genes during different developmental stages validated by qPCR in Chinese long ‘9930’

We investigated the expression of each *CLE* gene using published RNA-seq data (S5 Table) for 23 different cucumber tissues during vegetative and reproductive development. The expression level of each gene was normalized using the RPKM method. Ten cucumber *CLE* genes including *CsCLE1, 2*, *4*, *5*, *14, 25*, *24*, *23, 15*, and *17* showed relatively low levels of expression in all 23 samples, showing that they played a negligible role in developmental processes in cucumber. Genes *CsCLE9*, *10*, *18*, and *22* showed relatively high levels of expression across multiple samples (Fig. 7a).

Using qPCR, we analyzed the expression of cucumber *CLE* genes (S6 Table) in different tissues of Chinese long ‘9930’ seedlings. Out of 26 cucumber *CLE* genes, 24 genes were detected in 21 samples. Genes *CLE1*, *2*, *7*, *8*, *9*, *17, 18, 19,* and *26* were expressed at relatively low levels across all samples, and others were expressed at relatively high levels in at least one sample. Cucumber *CLE* genes showed relatively high levels of expression in the growing tissues, including point, stem, and flower, and relatively low levels of expression in the root. The expression level of *CsCLE4*, *6*, *11*, *14*, *21*, *23*, *24*, and *25* was the highest (Fig. 7b). The qPCR results are highly consistent with the former RNA-seq data available in the public database.

### Heterologous overexpression of *CsCLV3* gene in *Arabidopsis*

**Fig. 8:**
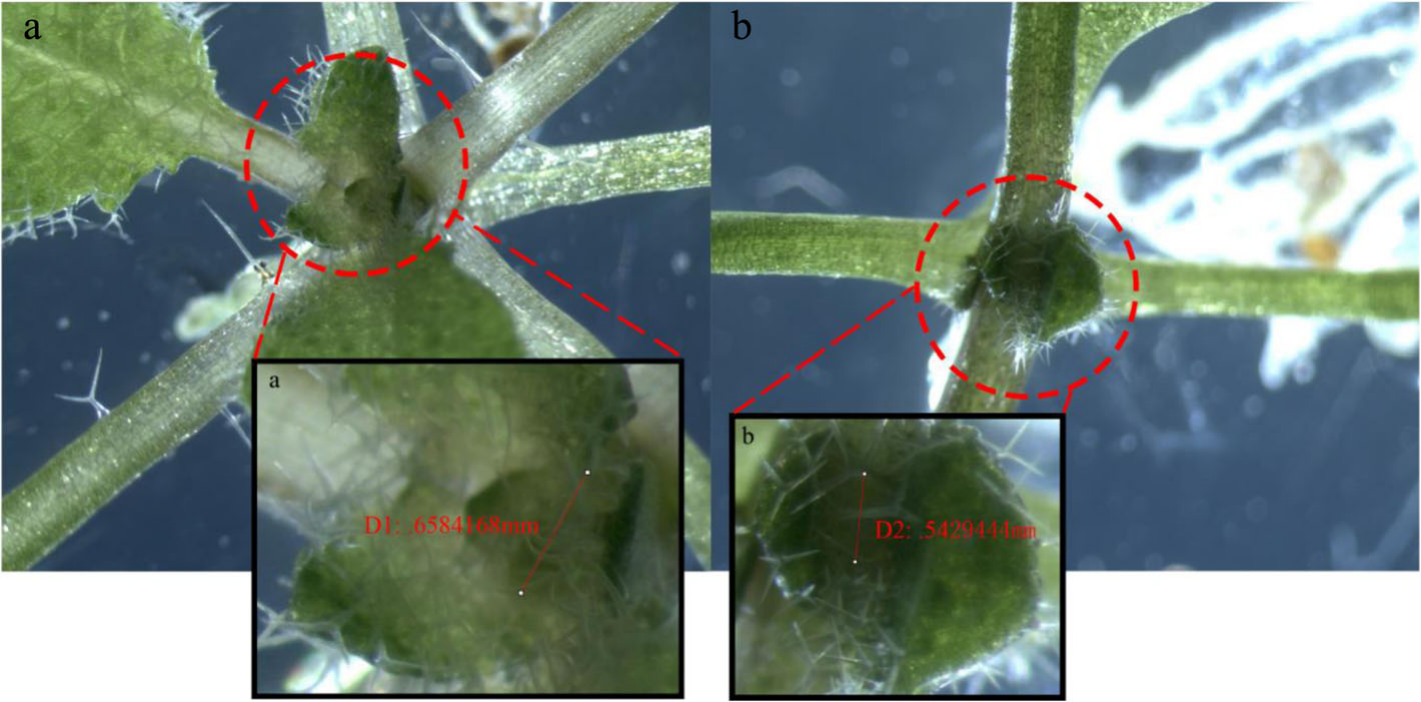
The growing point of heterologous overexpression of *CsCLV3* gene in *Arabidopsis*. **a** Growing point of wild type. **a** Diameter of growing point for wild type is 0.6584168 mm. **b** Growing point of positive strain. **b** Diameter of growing point for positive strain is 0.5429444 mm. The diameter of the growing point was measured with IPwin 32 software

The expression vector was successfully constructed by carrying the target gene on PHB plasmid, and then *CsCLV3* gene, the representative gene of the *CLE* family, was overexpressed in *Arabidopsis*; we refer to these transformed plants as positive strain. The results showed that in the phenotype of positive strain weakness developed at the growing point. Compared with wild type, the growing point diameter of wild type *Arabidopsis* was about 0.66 mm (Fig. 8a), and that of *CsCLV3* gene overexpression positive strain was about 0.54 mm (Fig. 8b). Under the same conditions, the growing point of the positive strain was smaller than that of the wild type.

**Fig. 9:**
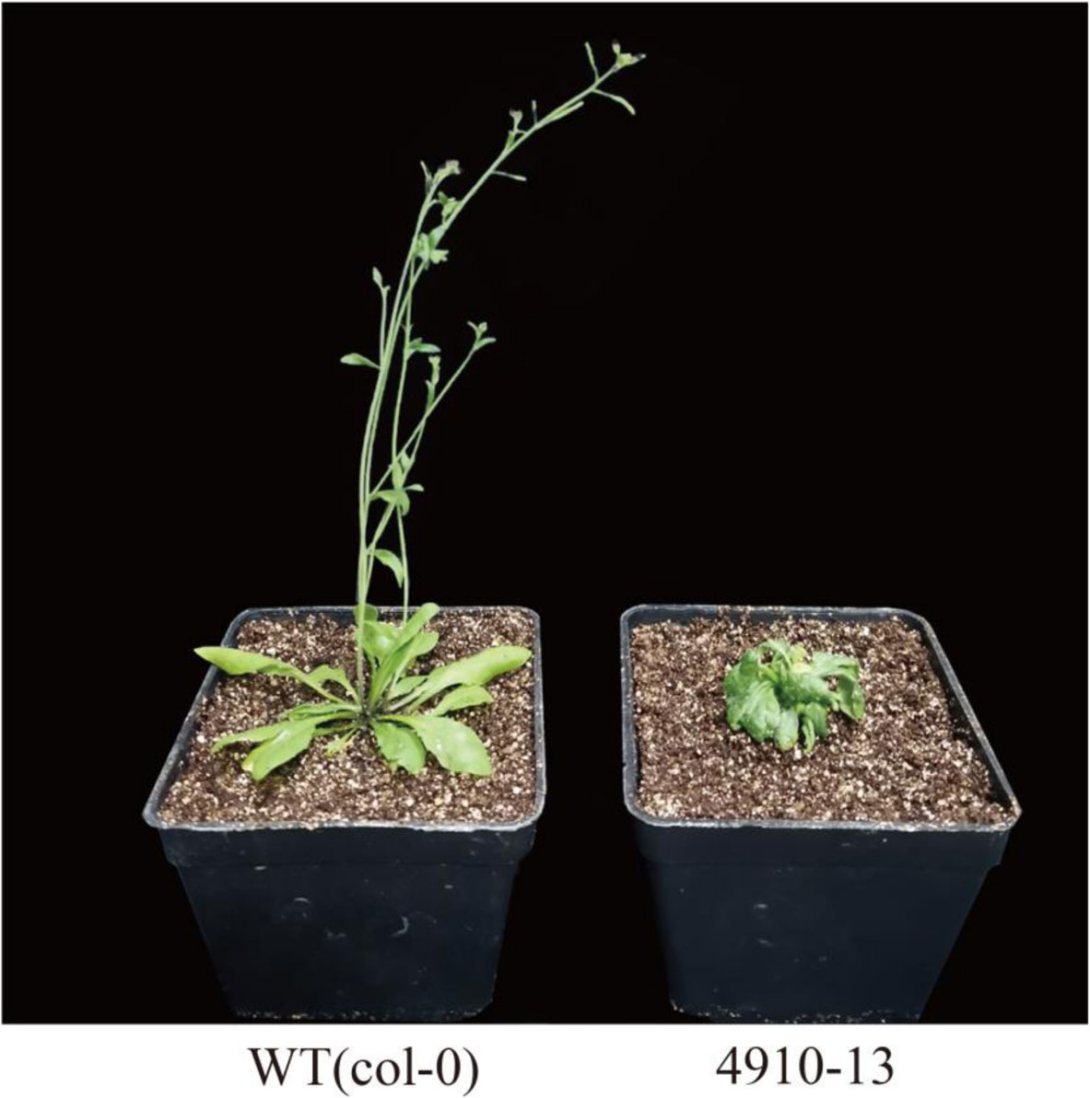
Heterologous overexpression of *CsCLV3* gene in *Arabidopsis*. Wild type (WT) strain has normal growth and development cycle, and phenotypic structure of rosette leaves, stems, leaves, pods and so on. The positive strain (4910–13) was the phenomenon of heterologous overexpression of *CsCLV3* gene, and it has no stem, leaf, fruit pod or other morphological structures, showing only wrinkled rosette leaves

**Fig. 10:**
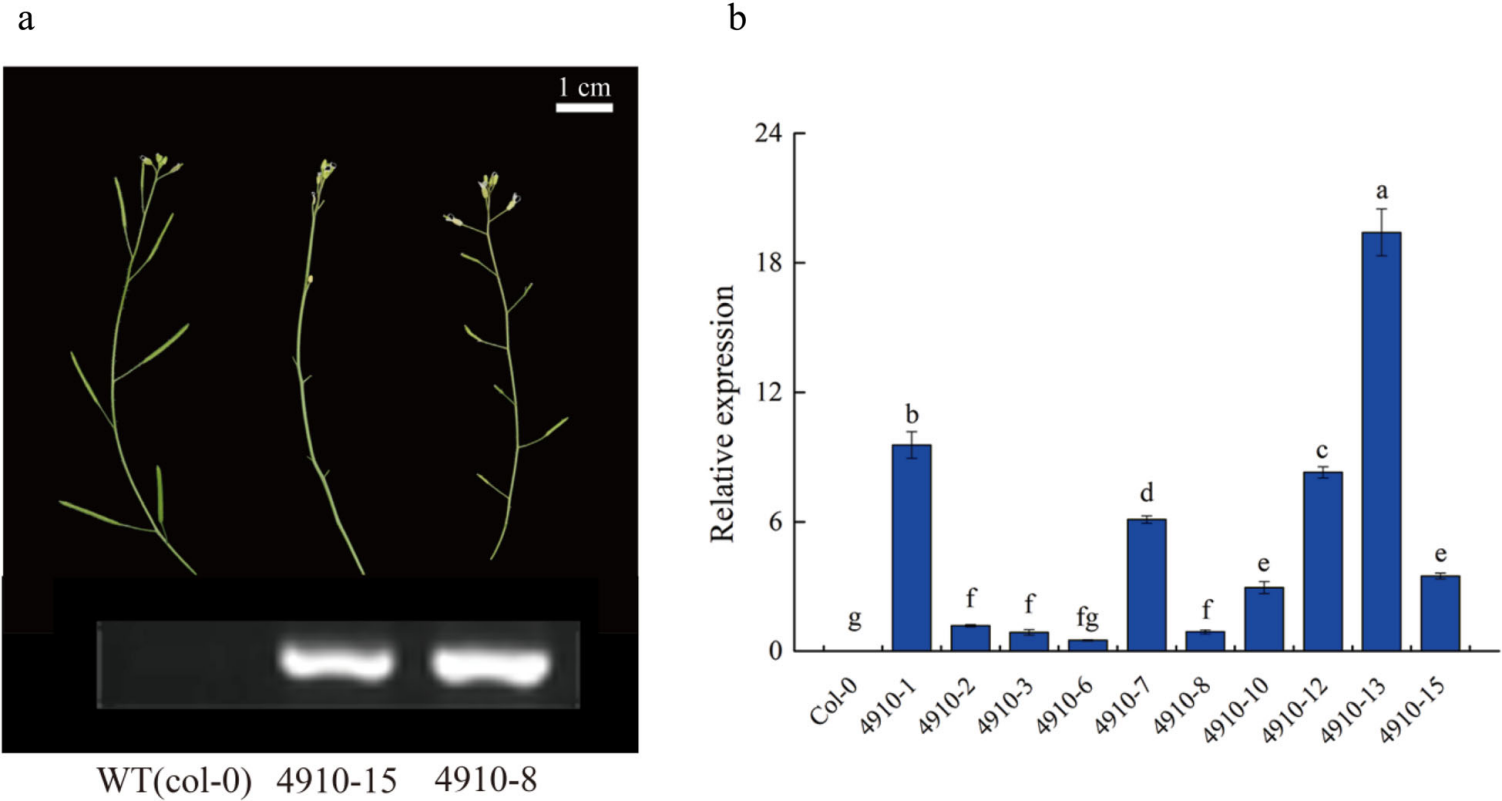
Pods of lower expression in positive strains. **a** Pod phenotypes of wild type (col-0) and positive stain (4910–15) could bolt but not bear fruit, while 4910–8 could bolt and bear fruit but internodes were shorter than in WT. Pod phenotypes of *Arabidopsis* were observed under a Nikon D3300 microscope. The bands shown in the figure are the gene expression corresponding to phenotypes. **b** Ten transformants were randomly selected for fluorescence quantitative PCR detection

After transplanting, almost all of the transformed plants in the T1 generation showed the phenomena of obvious weakness of the development of growing point, no bolting, and a decreasing capability of plant growth (Fig. 9). Heritable seeds obtained from T1 plants were extremely few, making functional analysis in subsequent generations unfeasible. Besides, few of the positive strains completed the processes of growth and development. In this study, only two bolted strains showed that either the pod did not develop or the pod was short, and its development was significantly inferior to that in the wild type (Fig. 10a). Ten transformants were randomly selected for fluorescence quantitative PCR detection (Fig. 10b). The results (S7 Table) showed that the target genes were expressed in all transgenic materials, and the phenotype of No. 15 and No. 8 with lower expression was the weakest, which could bear fruit but not great fruiting ability. At the same time, No.1, 12, and 13, which had high expression levels, had capable phenotypes and high degree of abnormality, and were unable to bolt or fructify. Generally speaking, the expression level of *CsCLV3* gene is consistent with its phenotype. The experimental results proved that cucumber *CLV3* genes function in a way essential in controlling the development of growing points and fruits, and plant bolting.

## Discussion

Our study investigated the CLE family genes in cucumber in detail. The spatiotemporal expression characteristics of all family genes in cucumber were also verified quantitatively. Gene *CsCLV3*, the representative gene of this family, was overexpressed in *Arabidopsis*. This work provides a new direction and idea for understanding the possible role of cucumber CLE proteins in plant growth and development, especially in fruit setting.

### Discovery of the *CLE* gene family in cucumber

A newly designed *CLE* Markov model was used in this study on the basis of which 26 new *CLE* genes were identified, proving that a CLE domain composed of 14 amino acids at the C-terminal is the conserved motif of the cucumber *CLE* gene family.

The replacement experiment of CLE domain in CLV3 revealed that the expression of fusion protein retained full physiological activity even after the upstream sequence of the CLE domain was replaced with an unrelated sequence [31]. The elimination experiment of variable domains in CLV3 demonstrated that the function of CLV3 is not significantly influenced by the absence of variable domains [32]. No MCLV3 activity was identified through the artificially synthesized CLE peptides lacking either Arg at the N-terminal or His at the C-terminal in the research of Kondo et al. [33]. In treating synthetic CLV3 peptides, Fiers et al. managed to totally inhibit the mutant phenotype of CLV3 [34]. Previous studies such as the abovementioned prove that the CLE domain is a key domain for the functioning of CLE peptides [35].

In this study, nearly every cucumber CLE protein containing motif 1 represented a typical CLE domain with CsCLE9 as an exception in which no typical CLE domain was detected. In maize, a CLE peptide expressed itself in primordial cells of organs but was manipulated and regulated by functional genes of FASCIATED EAR3 in stem cells, the regulatory function of which was achieved through leucine-rich-repeat receptors [36]. In the initial phase of flower development, stem cell niches are maintained through a negative feedback loop between the homeodomain transcription factor WUSCHEL (WUS) and the ligand-receptor system of CLAVATA3 (CLV3) [37]. The properties of stem cells and the size of FMs (floral meristem) are consequently affected when either of the genes above loses its activity [38]. The expression level published in both RNA-seq data and in the data of Chinese long ‘9930’ indicates a possible high expression of *CsCLE9* in tissues with high meristematic activity, which leads us to predict that *CsCLE9* may function in the CLAVATA–WUSCHEL feedback signaling between stem cells at the tip of the meristem and the underlying organizing center. This calls for further verification in later studies.

The 26 cucumber *CLE* family genes identified in this experiment exhibit a high degree of gene expression in meristem tissues, vascular bundles, and sites of vigorous growth as is shown in the analysis of public transcriptome data and real-time fluorescence quantitative analysis. However, only one of the 26 cucumber *CLE* family genes, i.e. *CsCLE6*, manifests a high degree of expression in plant roots, a finding inconsistent with those of previous studies. The tissue distribution of *CLE* genes influences their functional specificity to a large extent, as revealed in many tests of CLE protein ectopic expression at stem and root ends, but the location of gene expression is not the sole determinant of the functions of *CLE* genes [39], as shown in the cases of *CsCLE6* and *CsCLE9* in the current study. The CLE polypeptide family is a group of secreted peptide hormones that function among cells and play an essential role in the formation of stems, roots, vascular tissues, and pod nodules of plants [40, 41]. Numerous studies have demonstrated that CLE polypeptides are a significant kind of regulatory factors for keeping the balance between proliferation and differentiation of stem cells [34, 42].

### Phylogenetic analysis and evolution of cucumber *CLE* genes

According to the types of phylogenetic relationships, *CLE*s from three different plant species were divided in this research into seven groups within each group containing at least one *Arabidopsis* sequence. This provides necessary evidence for the identification of the conservation of *CLE*s in different plants. However, *CLE* genes do not distribute uniformly in the three different species. In cucumber, there are 12 *CLE* genes and the highest number of *Arabidopsis CLE* genes is found in Group 7, in which the largest number of *CsCLE* genes was identified. However, in neither Group 2 nor Group 6 were cucumber*-* or melon *CLE* genes found. Such difference may imply certain physiological significance. Rapid adaptive evolution is more likely to happen in groups with more genetic members than in groups with fewer genetic members, as hypothesized in a previous study [43]. In terms of the physiological functions of CLE peptides [44], the *Arabidopsis CLE* genes in Group 7 are classified as class A genes which function through the inhibition of the proliferation and division of apical meristem stem cells and root meristem stem cells. Cucumber and melon *CLE* genes that are classified into the same group can both be analyzed with reference to similar functions, the finding of which lays a foundation for future researches on the gene functions of *CLE* in Cucurbitaceae.

**Fig. 11:**
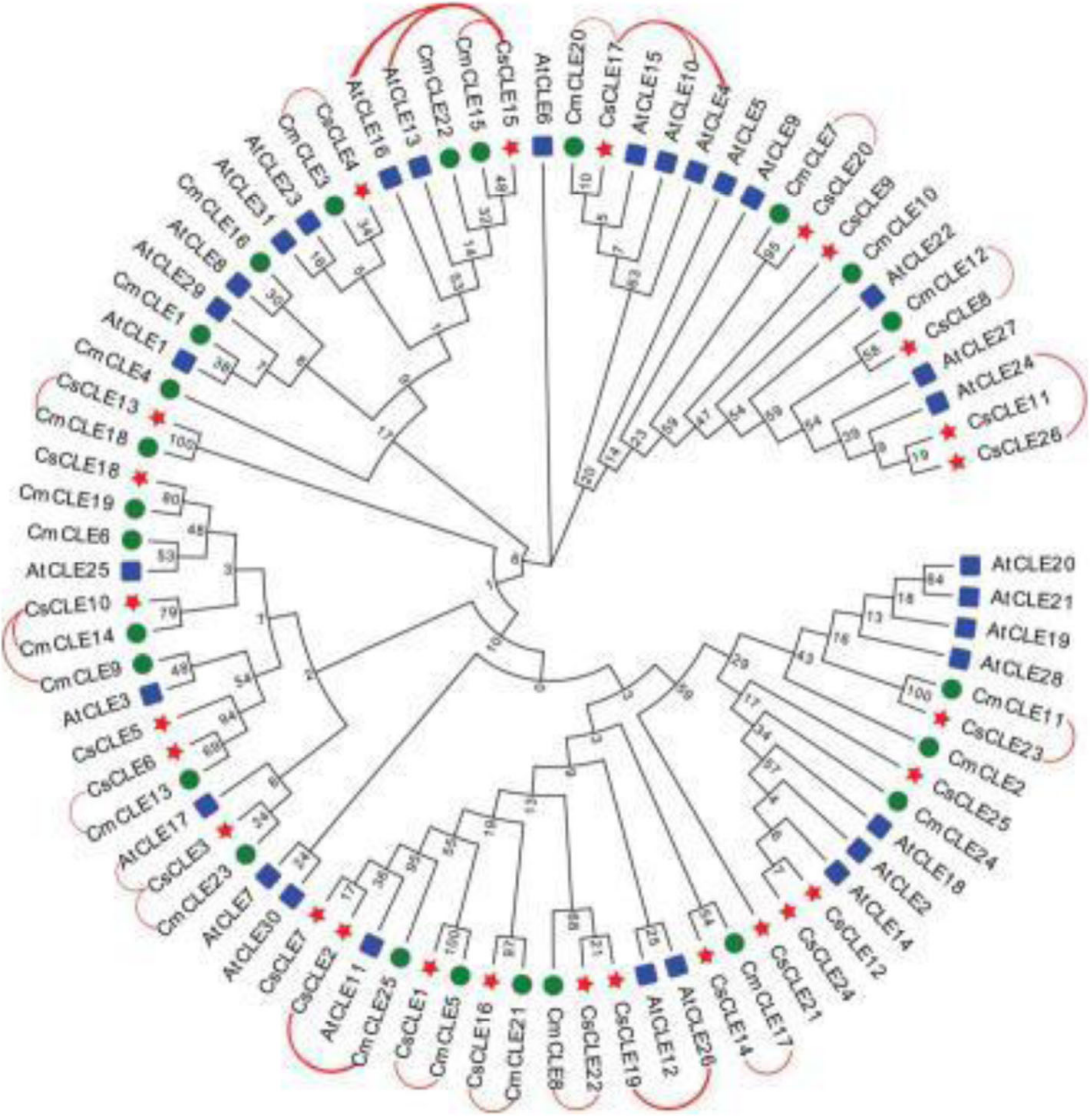
Comparison between the evolutionary and the species relationship, and the relationship among orthologous genes in *Arabidopsis*, melon and cucumber. The figure is the result of representing the homologous pairs in Fig. 6 in Fig. 3, and the red lines represent the homologous pairs of the *CLE* gene family

As shown in Fig. 11, we compared the evolutionary relationship and the species relationship and obtained the relationship among orthologous genes. It can be seen that the positions of orthologous genes in the phylogenetic tree are quite close, which indicates that the evolutionary relationship of *CLE* family genes among the three species is relatively close. Based on this, we can infer the functions of the related genes of cucumber and melon that are orthologous to those in *Arabidopsis*, as the latter has been studied in a relatively thorough manner. If *CsCLE19* is orthologous to *AtCLE26*, it can be inferred that its function is similar to *AtCLE26*; *AtCLE26* can inhibit the division of stem cells in the apical meristem and root meristem but cannot inhibit the division and differentiation of the procambium nor can it inhibit the differentiation of primary xylem vessels. This predicted function is different from the predictions of several other orthologous gene pairs, namely, *CsCLE3*/*CmCLE23*/*AtCLE17*, *CsCLE15*/*CmCLE15*/*AtCLE13*/*AtCLE16* and *CsCLE17*/*CmCLE20*/*AtCLE10*, which can inhibit the stem cells of apical meristem and root meristem. This difference can also be verified by measuring the gene expression. Gene *CsCLE19* exhibited a significantly different temporal and spatial expression pattern from *CsCLE15* and *CsCLE17*. In terms of the expression in the non-stem apex and root tip meristems, *CsCLE19* also had a higher expression.

Considering that a homologous gene of *CsCLE26* was found in *Arabidopsis* but not in melon, it is assumed that *CsCLE26* was retained in ancient cucurbits but was eliminated in melon in the process of evolution.

### Putative functions of *CsCLE* genes in hormone response and plant growth

The identification of cis-regulatory elements such as ABRE, AuxRR-core, and TCA-elements in response to hormones in 26 *CsCLE* genes indicated a dynamic mutual interaction between CsCLE polypeptides and hormones of other kinds. The frequent occurrence of cis-regulatory elements in either ABRE or ABA-dependent response uncovers the function of *CLE* genes in ABA dependent reactions under stringent conditions [45]. The gene name of *CLE* is a compound derived from the name of *CLV3* genes of *Arabidopsis* and that of Embryo Surrounding Region (*ESR*) genes of maize [46]. All the *CLE*s identified up to date share quite similar protein structures [47], usually with a short secretory signal peptide at the N-terminal, a long variable domain in between, and a conserved CLE domain at the C-terminal of the protein. Such conserved CLE domain is generally composed of 12–13 amino acid residues and is crucial for the functional execution of CLE proteins [48, 49]. One of the main features of *CLE* genes is their highly conserved structural domain at the C-terminals. Moreover, it is impossible to identify those *CLE* members that are expressed at relatively low levels using high-throughput sequencing. At the early stage of gene studies, the genome-wide identification of *CLE* genes in plants was conducted using BLASTP. Hidden Markov models and position-specific iterative BLAST method were employed by Oelkers et al. and more than 100 new *CLE* members were identified in 19 different species [46].

Essential elements such as the CAT-box and others that are crucial for *CLE* gene regulation in the process of maintenance of plant meristem and organogenesis, were definitely detected. Besides the analysis of the effect of overexpression of *Raphanus sativus CLEs* encoded with CLE peptides on secondary root structure in *Raphanus sativus*, Gancheva et al. [50] investigated the effect of synthetic CLE peptides treatment on *Raphanus sativus* and *R. raphanistrum* plants. For plants with a changing quantity of CLE peptides in roots resulting from either *CLE* overexpression or exogenous CLE treatment, the development of tissues in store roots alters accordingly. Additionally, a group of essential elements exist in numerous light regulation genes and groups of essential elements relate to environmental stimuli include responses to biotic and abiotic stresses. The existence of all four kinds of essential elements discussed above unveils the possible functions of *CLE* genes in corresponding responsive mechanisms.

### Cucumber *CLV3* genes act as critical regulatory in cucumber bolting and fruit bearing

Overexpression of *CLE* genes often shows a common dwarf and short-root phenotype [51], but in this study the overexpression of *CsCLV3* gene showed internode shortening but no obvious dwarfism in total stem length. Brand et al. [34] used transgenic plants overexpressing *CLV3* to show that the shoot meristem arrests after initiation of the first leaves (arrow), and the phenotypic results were similar to those in this study. Stamens and carpels are missing in the flower of the transgenic, at the same time, they also inspected the phenotype of clv3 mutants, and the mutant flower showed organ number increased particularly carpel. *CLV3* is a specific regulator of shoot and floral meristem development [52]. In *Brassica rapa*, it was proven that the multilocular mutant contained more stamens and carpels in the functional characterization of the multilocular silique gene *BrCLV3*, and most of its siliques had 4 locules with a shorter, rounder and thicker shape and extra gynoecium inside [53]. Knockout of rapeseed homologues of *CLV3* using the CRISPR/Cas9 system, and the double mutation of *BnCLV3* produced more leaves and multilocular siliques with a significantly higher number of seeds per silique and a higher seed weight than the wild-type and single mutant plants [54]. The gene of *CsCLV3*, a homolog of the *Arabidopsis* gene *CLV3*. Fine genetic mapping in F2 and RIL populations and association analysis in natural populations confirmed *CsCLV3* as the candidate gene for Cn (carpel gene) [55]. However, as for the *paeonia rockii* carpel, recent genetic studies [56] have shown that the phenotypic carpel is the complex of gene regulation networks. There are multiple candidate genes, and we are also actively verifying CLV3 as a candidate gene for cucumber carpel numbers. It is believed that the specific relationship between the CSCLV3 gene and cucumber carpel number phenotype will be clearly depicted in the near future.

The phenotype of weak growth point and loss of bolting and fruit bearing ability was obvious in the T1 generation. Our experiment of heterologous overexpression of representative *CLV3* gene in *Arabidopsis* revealed that more than half the number of transformed light-demanding plants in the T1 generation manifested responses such as stunted growth points, lack of bolting, plant shrinkage, etc., and some bolted lines grew to be short and stunted strains that were even significantly less developed compared to the wild-type strains. In contrast, the double mutation of *BnCLV3* produced more leaves and multilocular siliques with a significantly higher number of seeds per contributing to increased seed production. Results above demonstrate that *CsCLV3* gene function as a regulatory factor, most likely controlling plant development at different growing points and the processes of bolting and fruit bearing.

## Conclusions

Twenty-six *CLE* family genes were identified in the genome of Chinese long ‘9930’ cucumber. Almost all of the family members contained motif 1, a typical CLE domain, at the C-terminal. Analysis of CREs in the upstream promoter region of the genes predicted that this gene family might be related to hormone response in cucumber growth and development. The results of interspecies evolutionary analysis showed that the genes of the *CLE* family in cucumber were closely related to the model plants *Arabidopsis* and congener melon, with cucumber sharing seven orthologous *CLE* genes with *Arabidopsis* and 15 with melon. The analysis of a pair of orthologous genes in cucumber showed that as a part of the evolutionary process, *CLE* genes are undergoing a positive selection process which leads to functional differentiation. The family representative gene *CsCLV3* acts as a significant factor in the process of the development of growth points and functions in the growth of plant bolting and pod of those plants of positive transformation in which the expression was relatively low.

## Methods

### Identification of cucumber *CLE* genes

The genome, gene sequences, and corresponding protein sequences of cucumber were downloaded from cucumber genome database (CuGenDB,http://cucurbitgenomics.org/). The Markov models of two identical domains for the *CLE* genes were constructed using known *Arabidopsis* CLE proteins. All cucumber proteins were aligned with these Markov models using the HMMER program (http://www.hmmer.org/) with a domain cut-off E-value of e^− 3^. The *CLE* genes located at a distance of 10 kb apart on the same chromosome or scaffold were considered as tandemly duplicated genes.

The genome and annotated proteins of the model plant *Arabidopsis* were downloaded from TAIR database (https://www.arabidopsis.org/) [57]. The genome and annotated proteins of *Cucumis melo* were downloaded from CuGenDB database. Candidate proteins with only the CLE domains were eliminated manually. The Protparam (http://web.expasy.org/protparam/) [58] was employed to analyze the physical and chemical characteristics, including the molecular weight, theoretical isoelectric point (pI), atomic composition formula, instability index, aliphatic index, and grand average of hydropathicity (GRAVY), of the CLE proteins in these analyzed species.

### Phylogenetic analysis

The putative CLEs were determined by searching the melon genome database with HMM (Hidden Markov Model) profiles constructed using CLE sequences from Arabidopsis as queries. All the putative CLEs were further subjected to Pfam and SMART analyses to identify their conserved domains and signature sequences. Protein sequences from multiple species were used for a phylogenetic analysis *in planta*. Pairwise alignment and multiple alignment were performed using the ClustalX2 program with Gonnet protein weight matrix [59], and a maximum likelihood phylogenetic tree was built with the MEGA program (v6.06) using the conserved protein sequence of the cucumber *CLE* genes. Uniform rates and homogeneous lineages were adopted, and the partial deletion with a site coverage cutoff of 70% was used for gaps/missing data treatment. The frequency of each divergent branch higher than 50% was displayed. The figure was processed using the Adobe Illustrator software.

### Gene structure and motif analysis

The gene structure was analyzed using Gene Structure Display Server tool (http://gsds.cbi.pku.edu.cn/, v2.0) [60]. The MEME software (http://meme.nbcr.net/meme/, v4.12.0) was used to search for motifs (10 to 100 bp long) in the proteins [61]. Only widely distributed motifs that occurred in at least three protein sequences were retained. These motifs are represented in two separate figures, in accordance with the phylogenetic trees. The top ten motifs (with the lowest E-values) were reported, and statistically significant (lower E-value) motifs came first in the display.

### Upstream Cis-regulatory elements (CREs)

A 1500 bp fragment located before the initiation codon ATG of *CLE* gene family was captured use perl script from the cucumber genome sequence, and the online website PlantCARE (http://bioinformatics.psb.ugent.be/webtools/plantcare/html/) was used to analyze CREs of them. The figure was drawn using the online software GSDS (http://gsds.cbi.pku.edu.cn/).

### Identification of orthologous and paralogous genes

OrthoMCL software (v2.0.3) [62] was employed to search for orthologous, co-orthologous, and paralogous genes in *Arabidopsis*, cucumber, and melon using entire *CLE* protein sequences. An E-value cut-off of an all-against-all BLASTP alignment process was set at 1e^− 5^, and the alignment with a match cut-off value lower than 50 was eliminated. Nonsynonymous (amino acid-replacing, Ka) and synonymous (Ks) substitution rates among protein-coding sequences were used to reveal the DNA sequence evolution. The Ka/Ks ratio (Ka/Ks) was used to estimate the selective strength for DNA sequence evolution; Ka/Ks > 1 indicates positive selection, Ka/Ks < 1 indicates purifying (negative) selection, and Ka/Ks close to 1 indicates neutral mutation [63]. The ParaAT software (http://cbb.big.ac.cn/software) is capable of constructing multiple protein-coding DNA alignments in parallel for a large number of homologous genes [64]. To evaluate the divergence of duplicated cucumber *CLE* genes, synonymous rate (Ks), nonsynonymous rate (Ka), and evolutionary constraint (Ka/Ks) between paralogous pairs of genes were calculated with the KaKs_calculator tool and paraAT software using the method developed by Nei and Gojobori (http://cbb.big.ac.cn/software). The divergence time was calculated for the homologous pair using the formula T = Ks/2R, where R is the rate of divergence for nuclear genes from plants, and is R = 1.5 × 10^− 8^ synonymous substitutions per site per year for dicotyledonous plants [65]. The selected homologous pairs of *Arabidopsis*, cucumber, and melon were gathered and displayed using Cytoscape software (http://www.cytoscape.org, v2.8.3) [66].

### Tissue-specific gene expression analysis of *CsCLEs*

The gene expression data of cucumber *CLE* genes were gathered from CuGenDB (http://cucurbitgenomics.org/rnaseq/cu/17) (NCBI accession: PRJNA312872) [67]. The RNA-seq data from Cotyledon_4Week_Seedlings, FemaleFlower, Flesh_2Week_Fruit, Flesh_3Week_Fruit, Flesh_UnfertilizedOvary, FleshWeek_Fruit, Hypocotyl_4Week_Seedlings, MaleFlower_Bud, MaleFlower, OldLeaf, Peel_2Week_Fruit, Peel_3Week_Fruit, Peel_UnfertilizedOvary, PeelWeek_Fruit, Petiole_OldLeaf, Petiole_YoungLeaf, Root_4Week_Seedlings, Root Stem,Tendril, TrueLeaf_4Week_Seedlings, UnfertilizedOvary and YoungLeaf of cucumber cultivar Chinese long ‘9930’ were used. The TopHat program (https://ccb.jhu.edu/software/tophat/ index.shtml, v2.1.0) was used to map the reads to cucumber genome; expression profile of all the cucumber genes was then obtained with the FPKM (Fragments Per Kilobase of exon per million fragments Mapped) value using Cufflinks software (http://cole-trapnell-lab.github.io/cufflinks, v2.2.1) under the guidance of annotated gene models with a GFF file. The cucumber *CLE* gene expression profile from each sample was analyzed using the HemI program. The gene expression profile in each sample was standardized using RPKM (reads per kilobase per million reads) method. The expression profile of cucumber *CLE* genes from each sample was clustered and the heatmap was drawn using the HemI program (http://hemi.biocuckoo.org/). After normalization using the default linear method, expression data were clustered using hierarchical average linkage algorithm and Euclidean distance similarity metric algorithm in both the horizontal axis and the vertical axis.

### Tissue-specific gene expression in the cucumber line Chinese long ‘9930’

The cucumber line Chinese long ‘9930’ was used as the plant material to explore *CsCLE* expression differentiation in different tissues. Seeds were soaked for 15 min in water at 55 °C and placed overnight at 28 °C for germination. The seedlings were allowed to grow in a controlled-environment growth chamber programmed for a photoperiod of 16/8 h (light/dark) and an air temperature of 28/18 °C (day/night). At the four-leaf stage, roots, stems, mature leaves, young leaves, growth points, and cotyledons were harvested and snap-frozen in liquid nitrogen. At the flowering stage, flowers were harvested and snap-frozen before storage at − 80 °C for further analysis.

An RNA kit (Takara, Kyoto, Japan) was used to extract total RNA from the isolated plant tissues according to the manufacturer’s instructions. The Prime Script RT reagent kit (Takara, Kyoto, Japan) was used to reverse transcribe RNA into cDNA. The Primer 5.0 software was used to design specific primers according to the *CLE* gene sequences. Cucumber actin gene (CuGenDB name: Csa6G484600) was used as an internal control to normalize the expression level of the target genes among different samples. Three biological and three technical replicates were used. Each reaction was performed in a 20 μL reaction mixture containing diluted cDNA sample as the template, SYBR Pre-mix Ex Taq (2×) (Takara), and gene-specific primers. Quantitative real-time PCR (qPCR) was performed using a CFX96 Single color Real-Time PCR Detection System (BioRad, Hercules, CA, USA) with the following cycling profile: 95 °C for 20 s followed by 40 cycles at 95 °C for 3 s and 60 °C for 30 s; and a melting curve (61 cycles at 65 °C for 10 s) was generated to check the specificity of the reaction. The comparative Ct value method was employed to analyze the relative gene expression level. The RNA level was expressed relative to the actin gene expression level following 2^−∆∆CT^ method. The *CLE* gene expression cluster was analyzed via the TBTOOLS software [68].

### Heterologous overexpression of *CsCLV3* gene in *Arabidopsis*

The gene name of *CLE* is composed of the *CLV3* gene of Arabidopsis and the *Emeryo Surrounding Region (ESR)* gene of maize. In this experiment, the representative gene *CsCLV3* of the *CLE* family was heterologously overexpressed in *Arabidopsis* to explore the possible gene function of *CsCLV3*. Variety ‘GY14’ was employed as test material to clone *CsCLV3* gene, and its cDNA was obtained by the same method as for that of Chinese long ‘9930’ as mentioned above. The reference sequence was obtained from the cucurbit genomics database (CuGenDB, http://cucurbitgenomics.org/feature/gene/CsGy1G014910). The primers were designed by SnapGene, and the target gene was amplified. The gene cloning product and expression vector PHB were transformed into *E. coli* DH5α competent cells respectively, digested by double digestion method (the digestion sites were *Hind* III and *Xba* I), ligated by T4DNA ligase, cultured overnight at 37 °C, and the positive clones were screened by bacterial PCR. The expression vector with the target gene correctly loaded as a result of sequencing was obtained.

Transgenic plants of *Arabidopsis* were obtained by floral dip. The connected PHB expression vector was transferred into the competent cells of *Agrobacterium tumefaciens* GV3101 by freeze-thaw method. The bacterial suspension was prepared by the identified *A. tumefaciens* monoclonal using transformation buffer, and was used to infect plants of *Arabidopsis* when the OD value was about 1.0. Water was poured on the flowering plants 1 day in advance, and all inflorescences were inverted into the bacterial liquid suspended in advance by the transformation buffer for about 30 s; the transformation as repeated once after 7 days. After growing for 2–3 weeks, less nutrient solution was poured to accelerate aging, and mature seeds were harvested and dried for later determinations. Half-strength MS (murashige and skoog medium) (pH 5.8) solid medium with 30 μg mL^− 1^ hygromycin was used to screen positive seedlings of transformed plants. After disinfection, the seeds were sown on the screening medium, placed at 4 °C for 48 h, and then transferred to an incubator. The conditions for germination and growth were 25 °C, 16 h/8 h (light/dark), 30,000 lx light intensity, and 60% relative humidity. Positive strains could be distinguished after about 2 weeks, when they were transplanted and followed up.

## Supplementary Information

**Additional file 1: S1 Table**. Location and sequences of cucumber *CLE* genes. **S2 Table**. Location and sequences of *Arabidopsis CLE* genes. **S3 Table**. Location and sequences of melon *CLE* genes. **S4 Table**. Ka, Ks, and divergence time of the homologous pair *CsCLE24-CsCLE25*. **S5 Table**. RPKM of cucumber *CLE* genes during different developmental stages. **S6 Table**. Expression of cucumber *CLE* genes during different tissue in Chinese long ‘9930’. **S7 Table**. Expression of heterologous overexpression of CsCLV3 gene in *Arabidopsis*.

## Data Availability

The datasets supporting the conclusions of this article are available in the Treebase repository, [unique persistent identifier and hyperlink to datasets in http://purl.org/phylo/treebase/phylows/study/TB2:S27518?x-access-code=a142631fde4f4d2f9f68ba859afcbc38&format=html].
